# How to Improve Cord Blood Engraftment?

**DOI:** 10.3389/fmed.2016.00007

**Published:** 2016-02-17

**Authors:** Meral Beksac, Pinar Yurdakul

**Affiliations:** ^1^Department of Hematology, Ankara University School of Medicine, Ankara, Turkey; ^2^Cord Blood Bank, Ankara University School of Medicine, Ankara, Turkey

**Keywords:** cord blood, engraftment, cord blood transplantation, homing

## Abstract

Various factors make cord blood (CB) a significant source of hematopoietic stem cells (HSCs), including ease of procurement and lack of donor attrition, with the ability to process and store the donor cells long term. Importantly, high proliferative potential of the immature HSCs allows one log less use of cells compared to bone marrow or peripheral blood stem cells. As total nucleated cell (TNC) and CD34^+^ cell content of CB grafts are correlated to engraftment rate and speed, strategies to expand HSC and homing have been developed. This chapter will focus only on modalities such as intrabone administration, fucosylation, CD26 inhibition, prostaglandin E2 derivative or complement 3 exposure, and SDF-1/CXCR4/CXCL-12 pathway interventions that have been experimented successfully. Furthermore, increasing evidence in line with better recognition of CB progenitors that are involved in engraftment and homing will also be addressed.

The first decade of unrelated donor CBT (UCBT) experience was important in defining critical total nucleated cell (TNC) and CD34^+^ cell dose thresholds required for acceptable clinical outcomes, and in moving from related to UCBT and from pediatric to adult patients. The limitations of this approach also were defined during this period, with low cell dose identified as the critical barrier. Using double CB units (dUCBT) was a solution attempted by a group in Minnesota in 2000. Since then, the method has been proven to be safe and feasible. The advent of dUCBT brought a significant reduction in the risk of graft failure, opening up the possibility of HSCT with CB for essentially all patients without a suitable donor. Nonetheless, the use of dUCBT did not produce faster neutrophil recovery or immune reconstitution. Due to the high cost, it was limited to adult patients to reach the lower threshold. Thus, the advent of dUCBT has led to increased activity in the area of CB graft engineering, especially in the field of *ex vivo* expansion. Intrinsic and extrinsic cellular factors have been proven to act roles in hematopoietic stem cell (HSC) expansion, thus justifying their role in *in vitro* and *ex vivo* culture conditions. Attempts to regulate these factors through *ex vivo* expansion methods aim to overcome insufficient cell numbers. Delaney et al. achieved significant success justifying the role of triggering Notch-mediated signaling by Notch ligands (1, 2). Enhanced generation of CB hematopoietic stem and progenitor cells by culture with StemRegenin1 and Delta1 (Ext-IgG) was another *ex vivo* expansion approach from the same group (3). StemRegenin (HSC835) approach has later been undertaken by the Minnesota group and two Phase I/II clinical trials are ongoing (Clinical Trial Identifier: NCT01930162 and NCT01474681, respectively). In addition, Phase I/II clinical trials to evaluate the impact of infusion of expanded cryopreserved cord blood (CB) progenitor cells with Notch ligand or nicotinamide (NiCord) (Clinical Trial Identifier: NCT 01175785 and NCT01816230, respectively) on augmenting the UCBT outcome are ongoing in Fred Hutchinson and Duke Universities.

Apart from the expansion protocols applied for increasing cell dose, several other promising notions have also been introduced: optimal selection of HLA matching CB units, killer immunoglobulin-like receptor (KIR) typing of the candidate units; modification of conditioning as well as GVHD prophylaxis regimens; post-transplant use of growth factors/cytokines; and infusion of the CB with accessory mesenchymal stem cells (MSC) are of the most forthcoming modalities as nicely detailed in the recent review by Rocha et al. (4). Results of a clinical trial led by MD Anderson Cancer Center (Clinical Trial Identifier: NCT 00498316) for testing CB expansion on MSC has been addressed in this issue of the journal (5). Despite the fact that many different strategies have gained attention with various fold increases in CD34^+^ cell numbers, delayed immune reconstitution remains a major challenge after UCBT. There are currently many ongoing *in vitro*/*in vivo* experiments along with preclinical and clinical trials for the evaluation of different strategies for improving engraftment after UCBT (6–9). This review aims to summarize the most prominent approaches those having impact on HSC engraftment with the exception of double UCBTs as this approach has become an established transplant modality for patients who lack an HLA matched adult donor.

## Predictors of Engraftment

Generally, TNC and CD34^+^ are considered to be the best predictors of engraftment standard for selection of units. As discussed in detail in the review by Beksac and Preffer, quantification of HPSC capacity of a graft is still not standardized and differs according to the source of stem cells (10). In the CB, TNC contains a considerable number of normoblasts and lymphocytes. The marrow TNC is more heterogeneous. In CB, the CD34^+^ per microliter is almost as high as the counts following 3–4 days of G-CSF administration. Migliaccio et al. (11) have compared TNC with colony-forming unit (CFU) numbers and have shown TNC to be inferior to CFU in determining neutrophil and platelet recovery speed following UCBT. Recently, Simmons et al. have identified a CD34^+^ cell subpopulation that co-expresses an antigen (-MA6) (12). This antigen expression predicted platelet engraftment better than CD34^+^ cell counts. In this study comparison of PBSC with CB, it revealed less frequent MA6 expressing cells within CB (<0.2%) than PBSC (8%) CD34^+^ cells. This finding provides an explanation to slower platelet recovery following single UCBTs. The impact of megakaryocytic lineage commitment within CD34^+^ cells following *in vitro* manipulation of CB is not known yet.

## Intrabone Infusion

Historically, the earliest HSCT experience was performed using bone marrow (BM) with direct intrabone infusion. As this approach required technical expertise and induced pain in the recipient, it was later replaced by central venous administration through catheters. Frassoni et al. were the pioneers to revisit intrabone application in UCBT (13). Their preliminary results on 44 patients were able to show neutrophil and platelet recovery times to be 23 and 36 days, respectively. Full donor type chimerism was obtained among all patients. This success tempted investigators to perform a retrospective registry-based analysis, to compare outcomes of results from intrabone single IB-UCBT (n:87) vs. dUCBT (n:149) after myeloablative conditioning regimen (4). Although the median number of TNC were lower among the IB-UCBT (2.5 × 10 vs. 3.9 × 10/kg, *P* < 0.001), neutrophil recovery (76 vs. 62%, *P* = 0.014), median time to engraftment (23 and 28 days, *P* = 0.001), and platelet recovery (74 vs. 64%, *P* = 0.003) were better. In multivariate analysis, IB-UCBT was associated with higher neutrophil and platelet recovery and lower acute graft-versus-host disease (II-IV) (*P* < 0.01). IB-UCBT did not increase TRM and there was a trend for longer PFS.

## Enhancing Bone Marrow Homing

Full recovery of tri-lineage hematopoiesis following stem cell transplantation depends on proper engraftment of transplanted cells, which relies on engraftment of the most primitive long-term repopulating HSCs (1) To overcome the negating effects of low counts of HSC within CBUs, strategies to improve homing of HSCs to the marrow have been developed. Recently, two new strategies have been proposed. The first is based on inhibition of human analog of the murine-identified HSC-expressed dipeptidyl-peptidase (CD26). The second, developed by Minnesota group, is based on *ex vivo* priming of HSCs before transplantation with small molecules, such as C3 complement fragments, fibrinogen, fibronectin, and hyaluronic acid. This second strategy has been adopted by other groups, such as the investigators in Brigham’s Hospital, who have used a prostaglandin E2 derivative to facilitate homing and induce hematopoietic progenitor proliferation (14).

### Effects of CD26 and Modification of CXCR4-SDF-1 Axis

SDF-1, a BM stromal cytokine, plays a significant role in homing of HSCs in BM niche through binding its receptor CXCR-4. It has been suggested that retention of CB cells in the BM depends highly on the level and SDF-1 binding capacity of CXCR4 after transplantation and allogeneic engraftment (15). Moreover, an efficient SDF-1 gradient and adequate responsiveness of CXCR-4 receptors to SDF-1 in the BM microenvironment are proven to be one of the main cellular events providing engraftment of HSCs in BM (16, 17). Through interaction between cells expressing CXCR4 and inflammatory chemotactic molecules, matrix metalloproteinases (MMPs), and angiopoietic factors (e.g., VEGF) SDF-1/CXCR4 axis plays a crucial role by increasing seeding efficiency and speed (1, 17).

The Indianapolis team led by Broxmeyer was the first to discover inhibition or deletion of CD26 on donor cells to enhance short-term homing, long-term engraftment, competitive repopulation, secondary transplantation, and mouse survival in an experimental model. Their pivotal findings suggest that CD26 is a novel target for increasing transplantation efficiency. The peptidase CD26 [DPPIV (dipeptidylpeptidase IV)] removes dipeptides from the amino terminus of proteins. They provided evidence that endogenous CD26 expression on donor cells negatively regulated homing and engraftment. CD26 is a widely expressed membrane-bound ectopeptidase that cleaves CXCL12, thereby depleting its chemokine activity and explains the homing/engraftment effects. Based on this fact, inhibition of the enzyme (CD26/DPPIV) cleaving SDF-1/CXCL-12 (CXCR4) into a truncated form has been a promising approach to retain the hindered chemotactic effect.

Many different researchers have also provided *in vitro* and *in vivo* data that inhibition of CD26/DPPIV clearly enhanced colony stimulating activity as well as engraftment capacity of CB HSCs (17, 18). The clinical efficacy of a DPP-IV inhibitor (Sitagliptin), FDA approved for treatment of type II diabetes mellitus, is under investigation in two clinical Phase II studies. Led by Sherif Farag, authors observed a speed up and enhancement of engraftment following CB transplantation (Clinical Trial Identifiers: NCT 00862719 and NCT 01720264) (19). Among 24 patients following a MAC regimen and oral 600 mg Sitagliptin (−1 to +2 days) suppressed DPP4 activity by ~70–80%, although the effect was not permanent with a return to baseline activity levels within 16 h of administration. A key finding was a significant correlation between level of DPP4 suppression and neutrophil engraftment (*P* = 0.002). However, the optimal Sitagliptin dose as well as the duration needs to be further investigated clinically (9, 19).

SDF-1 regulates the trafficking of pre-B lymphocytes and T lymphocytes in addition to CD34^+^ HSCs. Thus, modulation of cell trafficking by SDF-1, particularly *via* adhesion molecules, also happens to effect survival of CB T cells in BM environment. These data justifies CXCR4-SDF-1 axis’ role in homing and mediating of allogeneic CB engraftment of cells that need to combat recipient immune-mediated graft rejection (15).

Recently, the Leuven group led by C. Verfaille identified a new inhibitor: tissue factor pathway inhibitor (TFPI) that acts as a biological CD26 inhibitor on human marrow or CB hematopoietic progenitor and stem cells. TFPI exerts activity through Glypican-3, which is co-expressed with CD26. Their experimental results have not entered clinical trials yet.

These results suggest that HSC engraftment is not absolute, as previously thought, and indicate that improvement of BM transplant efficiency may be made possible in the clinic.

### C3a–C3aR Axis

One of the major components of the innate immune system is the complement cascade proteins. Treatment with complement proteins, C3a in particular, has been shown to enhance *ex vivo* transmigration of CD34^+^ HSCs from CB and BM *via* elevating the expression of CXCR-4 and MMP-2/MMP-9. Short-term priming of CB-derived CD34^+^ HSCs may upregulate levels of homing-related molecules. Their *ex vivo* trans-migratory and *in vivo* homing potential may overcome the delayed reconstitution after UCB (1).

Ratajczak and co-workers showed that the complement cascade is activated during growth-factor-induced hematopoietic progenitor and HSCs mobilization and that complement cleavage fragments play a part in the mobilization. It was reported that the C3a receptor (C3aR) is expressed on CB CD34^+^ cells and that CD34^+^-cell migration toward SDF-1 could be enhanced through C3a *in vitro* (20). In addition, after pretreatment of CB CD34^+^ cells with a C3aR antagonist (SB290157) homing after transplantation into NOD/SCID mice was impaired (21, 22). In the Phase I clinical trial led by Brunstein et al., the effect of C3a priming was investigated following one primed and one unmanipulated CB among patients with high-risk hematological malignancies (23). In this double CB platform, median time to engraftment was 6 days, which was not significantly different from the time to engraftment of historical non-myeloablative UCBT control patients. Engraftment was dominated by the C3a-primed CBU among nine of 27 evaluable patients. Engraftment was driven by the CD3^+^-cell content of the CBU. CD3^+^ more than 0.5 × 10^7^ cells/kg determined the winner (23).

### Modulation with Prostaglandin E2

The stable prostaglandin E2 derivative 16,16-dimethyl prostaglandin E2 (dmPGE2) has been identified as a critical regulator of HSC homeostasis following screening among many chemical molecules in a zebra fish embryo model (24). A brief incubation with dmPGE2 up-regulates the genes responsible from homing (e.g., CXCR4), proliferation (e.g., CyclinD1), and cell survival (e.g., Survivin) (14). Authors hypothesized that brief *ex vivo* modulation with dmPGE2 could improve patient outcomes by increasing the “effective dose” of HSCs. In a Phase I trial following a RIC dUCBT, they were able to achieve accelerated and long-term neutrophil engraftment (17.5 vs. 21 days, *P* = 0.045). The dominating CBUs were those exposed to dmPGE2 in 10/12 of CBTs. Furthermore, there were no adverse events or safety issues related to *ex vivo* dmPEG2 exposure. A Phase II clinical trial (NCT01627314) is underway to further investigate the feasibility and effectiveness of this manipulation. These authors have expanded their research to discover dmPEG2 priming to modify Wnt signaling resulting in T cell factor (TCF)-mediated transcription. Wnt signaling upregulated interleukin (IL)-7R and IL-2Rβ expression resulting in enhanced survival mediated by the homeostatic cytokines IL-7 and IL-15 (25). This novel approach induces and maintains naive, memory precursors, and long-lived central memory CD8^+^ cells. These immune-mediated effects are very promising as immune reconstitution is delayed following CBT and constitutes one of the major problems.

### Fucosylation

Delayed engraftment was shown to be due, at least in part, to low fucosylation (the addition of a fucose molecule) of cell surface molecules important for homing to the BM microenvironment. Interaction among adhesion molecules within the BM micro-niche depends on the fucosylation of CB HSC. A simple 30-min *ex vivo* incubation of CB hematopoietic progenitor cells with fucosyltransferase-VI and its substrate (guanosine diphosphate fucose) is sufficient to increase fucosylation (26, 27). Fucosylation has been shown to improve engraftment among irradiated NOD/SCID mice (4). Moreover, Robinson et al. demonstrated that only fucosylated CB CD34^+^ were responsible from engraftment among NOD-SCID interleukin-2Rγ (null) mice (28).

In MD Anderson Cancer Center, two Phase II clinical trials are underway to investigate the utility of CB CD34^+^ cell fucosylation (Clinical Trial Identifiers: NCT 01471067 and NCT 02423915, respectively). Expectations are to test clinical utility of fucosylated T cells with an aim to obtain faster engraftment and prevent GVHD among patients with leukemia–lymphoma. Encouraging results supporting *ex vivo* fucosylation in dUCBT setting of the registered NCT 1471067 trial was recently published by Popat et al. (27). Effects of fucosylation were evaluated among 22 patients enrolled in the trial. The median time to neutrophil engraftment was 17 days (range, 12–34 days) compared to 26 days (range, 11–48 days) observed in a control group of 31 patients who had undergone double unmanipulated UCBT (*P* = 0.0023), Platelet engraftment was also improved: median was 35 days (range, 18–100 days) compared with 45 days (range, 27–120 days) for controls.

## Co-Administration of CB-Derived Progenitor or Natural Killer Cells

Another strategy to improve engraftment has been based on co-administration of third party stem cells. Robin et al. have demonstrated human stem and progenitor cells are detectable in placental tissue (29). Celgene Cellular Therapeutics (CCT) has developed a proprietary, sterile closed perfusion process for the collection of human placenta-derived stem cells (HPDSCs) from full term placentas. HPDSC are rich in HSCs and hematopoietic progenitor cells, HSC and progenitor CFU forming capabilities, low in HLA Class I and II expression and enhance *in vivo* engraftment when combined with UCBT in NOD-SCID animals. Elmacken et al. have initiated a pilot study of adding universal donor (third party) HPDSCs with either single or double UCBT following myeloablative or reduced toxicity conditioning in children and adults with selected malignant and non-malignant diseases in a multicenter consortium (IND#14949; Clinical Trial Identifier NCT 01586455) (30). In their preliminary ASBMT 2015 abstract, they reported a median time to neutrophil engraftment of 22 days following a mean (±SD) cell dose of CD34^+^ (3.9 ± 1.8 × 10^5^/kg) and HPDSC CD34^+^ (0.3 ± 0.15 × 10^5^/kg) infusion.

The use of CB originated immune cells is one of the most promising immunotherapeutic strategies to be used after CBT (16, 31–33). Induction or adoptive transfer of CB-derived immune cells, particularly natural killer (NK) cells and regulatory T cells (T reg) with or without cytokines are also effective approaches for gaining better engraftment levels after UCBT. All of these approaches have been denoted as “successful” in preclinical *in vitro* and animal studies (34–36).

The role of NK cells in engraftment is still controversial. Gertow et al. have suggested that mixed chimerism following double UCBT could possibly be related to NK cell tolerance between the CB units; other reports, however, did not show a correlation between KIR ligand incompatibility and engraftment (37). Nonetheless, previous studies of HSC transplantation in mice demonstrated that IL-2-activated NK cells mediate HSC engraftment and that alloreactive NK cells may facilitate engraftment by killing recipient T cells and APCs. As the reduced function and maturation of NK cells arising in the early post-UCBT period can be restored by cytokines, infusion of *ex vivo* expanded and activated NK cells could represent a means to enhance early engraftment following UCBT.

## Conclusion

Until now, many expansion modalities with the ultimate goal of improving engraftment after UCBT were successful to a certain degree. It is of great interest that current evidence supports priming of CB cells prior to transplantation modulates/improves engraftment dynamics. In addition to generating increased numbers of progenitor cells with rapid *in vivo* re-population capacity, enhancing HSC homing capacities will improve the kinetics of hematopoietic recovery with better transplant outcome and hopefully less transplant related mortality rates.

## Author Contributions

Both authors contributed to the mini review in close collaboration.

## Conflict of Interest Statement

The authors declare that the research was conducted in the absence of any commercial or financial relationships that could be construed as a potential conflict of interest.
